# Integrated Project Scheduling and Staff Assignment with Controllable Processing Times

**DOI:** 10.1155/2014/924120

**Published:** 2014-04-24

**Authors:** Victor Fernandez-Viagas, Jose M. Framinan

**Affiliations:** Industrial Management, School of Engineering, University of Seville, Camino de los Descubrimientos, s/n, 41092 Seville, Spain

## Abstract

This paper addresses a decision problem related to simultaneously scheduling the tasks in a project and assigning the staff to these tasks, taking into account that a task can be performed only by employees with certain skills, and that the length of each task depends on the number of employees assigned. This type of problems usually appears in service companies, where both tasks scheduling and staff assignment are closely related. An integer programming model for the problem is proposed, together with some extensions to cope with different situations. Additionally, the advantages of the controllable processing times approach are compared with the fixed processing times. Due to the complexity of the integrated model, a simple GRASP algorithm is implemented in order to obtain good, approximate solutions in short computation times.

## 1. Introduction

The lead time required to carry out a project—project makespan—has turned to be one of the main sources of competitive advantage for companies (see, e.g., [1, 2]). Since, in many cases, the processing times of the tasks that compose a project depend on the resources allocated to the task (see, e.g., [3]), it is clear that the project lead time depends both on the scheduling of the tasks and on the allocation of staff to these tasks. Regarding task scheduling, it encompasses the sequence to be followed by each task over time, and it is usually regarded as an important aspect of the industrial organization [4] and production [5]. On the other hand, staff assignment is essential in a project [6] and it can be defined as the allocation of company workers to different tasks. Traditionally, staff assignment is included within the manpower planning as one of its steps. Thus, several authors [7–10] have considered the following structure: (1) planning; (2) scheduling; (3) allocation. In staff assignment, worker's satisfaction tends to be maximized whereas makespan and production costs are often minimized in task scheduling. To execute a task, it is essential that a particular employee is available at that time; otherwise the realization of such task should be postponed until the employee becomes available and, consequently, staff assignment directly influences task schedule. Due to the importance of the scheduling and the assignment in a project environment and since both are very related, it is critical to address both problems in an integrated manner. Furthermore, the majority of scheduling problems in the literature consider fixed processing times [11] and most of the rest assume that processing times depend linearly on the amount of resources assigned [12]. As lead times strongly depend on the processing times of the task, and since the processing time of a task depends on the number of employees assigned to this task, in this paper we explicitly take into account this relationship.

In this paper, an integration of project scheduling and staff assignment (PSSA) problem with controllable processing times (CPT) is presented using renewable resources and nonpreemptive tasks. Minimization of the makespan has been chosen as the objective function since delays can lead to an increase in costs and even to the loss of customers. This problem can be placed within the project management process. According to Demeulemeester and Herroelen [13], the project life cycle is as follows.

- *Concept Phase*. There is a need of a customer to perform a project. This need is transmitted to the company.
- *Definition Phase*. First, the goal of the project is defined. Next, the work content is defined and, finally, a project strategy is elaborated to achieve the goal.
- *Planning Phase*. The project is divided into tasks. Then, their processing times are estimated and both the resources requirements and the precedence relationships between tasks are identified.
- *Scheduling Phase*. Both task scheduling and the amount of resources in each period of time are defined in this phase
- *Control Phase*. In this phase it is controlled that the project is implemented following the aspects defined in the planning and scheduling phases.
- *Termination Phase*. This phase corresponds to the delivery of the results of the project.

Note that between the scheduling phase (where the amount of resources is known) and the control phase there must be an assignment of resources to the tasks if the resources are not identical. For these cases, an additional phase is therefore needed. This phase—named “resources assignment phase”—must be placed between the scheduling phase and the control phase. Alternatively, integration between the scheduling and the resources assignment phases can be done. The latter is the adopted approach in this paper, which is organized as follows: a state of the art and a description of the problem are shown in Section 2. An extended explanation of the CPT is also presented, while the problem statement and a formulation of the integer linear programming model are shown in Section 3. Additionally this integer linear programming model is compared with a multimode formulation. In Section 4, we define a simple GRASP heuristic algorithm for the problem.  Section 5 contains computational experiments based on a test bed. Besides, a comparison between the model with and without CPT is shown there. Lastly, conclusions are described in Section 6.

## 2. Literature Review

The model presented in this paper includes two decision problems: task scheduling and staff assignment. Task scheduling is traditionally denoted in the literature as* project scheduling problem* (PSP). Furthermore, if limitations regarding the number of resources per activity are assumed, it is named* resource-constraint project scheduling problem* (RCPSP). This problem has been extensively studied in the literature, and recent reviews can be found in Węglarz et al. [32] and Hartmann and Briskorn [33]. Staff assignment is a type of assignment decision problem that has also been extensively addressed [34]. Nevertheless, the integration between project scheduling and staff assignment has not been so comprehensively studied, and hence there is still no consensus about the name of this joint problem (see in Table 1 different names that have been used to denote the problem). In our work, we consider the name “project scheduling and staff assignment” as denomination for the problem.

Our problem is related to several contributions in the literature, which are summarized in Table 2. Bassett [29] presents a model of project scheduling and staff assignment considering time windows for the completion times of the tasks. Vairaktarakis [18] adds precedence relationships to the integrated problem. However, no time units are used in the linear programming model (the order of the tasks defines the schedule of the project). Time units are included in a similar problem by Bellenguez and Néron [14] and Bellenguez-Morineau and Néron [15] where some lower bounds are calculated in the former reference, while in the latter the authors implement a branch and bound algorithm. External and internal resources constraints are considered by Kolisch and Heimerl [24]. Wu and Sun [27] present the integrated problem proposing a nonlinear model, which is solved by a genetic algorithm. Although learning effect (i.e., the longer an employee works on a task, the greater his/her efficiency is) is considered in the model, neither precedence relationships nor skill constraints are used in their model. Gutjahr et al. [26] consider portfolio selection and skills in the integrated problem with learning effect but with precedence relationship which are included by Gutjahr et al. [25]. Precedence relationships are also included by Corominas et al. [20] in an integrated problem, similar to the problem of Wu and Sun [27], also with learning effect. Drezet and Billaut [17] propose a linear programming model for a project scheduling and staff assignment problem. However, they do not consider learning effects in the model and include move constraints (i.e., the number of assignment movements of the employees from one nonfinished task to another is bounded). Moreover, the number of employees assigned to each task at each period of time is set between a minimum and a maximum depending on the level of skills of the workers. However, the processing times of the tasks are assumed to be fixed and not depending on the number of employees assigned. Since no solutions can be found for medium-sized instances of the problem, a tabu search algorithm was implemented using a greedy algorithm as initial solution.

A common feature in all the aforementioned works is that none of them include variable processing times. Regarding CPT, Hachicha et al. [30] propose a staff assignment model considering multiskilled employees, preference of the employees for some tasks, and skill-based processing times (i.e., the processing times of the tasks depend on the skills of the employee). Nevertheless, they are preprocessed before solving the model, which means that the processing times do not change during the planning horizon. More specifically, processing times are first calculated depending on the skills of each employee and then introduced in the model as data. Heimerl and Kolisch [31] analyze a staff assignment problem without scheduling considering learning effect on the employees, so the processing times are now variables of the problem as they depend on the experience of the staff. In order to solve this problem, a nonlinear model is implemented. Hachicha et al. [30] and Heimerl and Kolisch [31] use CPT in their models, although none of them solve the integrated problem (only the staff assignment problem). Variable processing times in a scheduling problem can be found in Alfares and Bailey [28]. They use a linear programming model to schedule the task of a project and to assign the number of employees to each task; that is, the variables of the problem are the starting times of each task, the processing times, and the number of employees per period. Regarding the processing times, they are chosen to minimize the costs, but they do not depend on any variable. Moreover, staff assignment for each employee is not considered there.

CPT in an integrated PSSA problem are considered in Drexl [21], Dodin and Elimam [22] with the objective of costs minimization. However, in their models, the processing times depend on the skills of the employee assigned (and consequently are introduced in the model as data), but not on the amount of resources allocated; that is, processing times are defined by a matrix of two dimensions where the rows are the processing times of the tasks and the columns the employees. In Valls et al. [19], each task has to be performed by a single employee and its processing time increases or decreases depending on the efficiency of the employee. Nonpreemptive tasks with release times and due dates are taken into account, as well as precedence relations with time lags. No programming model is implemented, but a hybrid genetic algorithm is developed to solve the cost minimization problem. This contribution is the one most related to our problem, since the authors implement an integrated PSSA with CPT. However, in our paper, a linear programming model is considered to minimize the makespan of the project where the processing times of the tasks depend piecewise linearly on the number of employees assigned instead of the efficiency level of the employee and multiple assignments to each task are also allowed as well, while in Valls et al. [19] each task must be implemented by a single employee. To the best of our knowledge, the proposed model has not been analyzed before.

## 3. Model Proposal for the Integrated PSSA Problem

### 3.1. Problem Statement

This paper presents a PSSA problem in which a company is responsible for the implementation of a project consisting of *J* tasks (with task *j* = 1,…, *J*) with (known) precedence relations between tasks (i.e., some tasks should be finished before carrying out others). The order of execution of each task has to be decided to minimize the corresponding objective function. Each task must be performed by some workers from total of *E* employees (employee *e* = 1,…, *E*) with certain skills in each *t* period (*t* = 1,…, *T*) of the planning horizon *T*.

It is assumed that each task *j* has a release time *r*_*j*_, so it must always start after this time. Furthermore, once a task starts, it runs continuously until its end (nonpreemptive approach). One employee can be assigned to a task if the employee possesses the required skills for the task. We assume that there is an optimal number of employees, *R*_*j*_, to be assigned to each task, although overcoverage and undercoverage of employees are allowed by the model. Under- and overcoverage would lead to different values of the efficiency of the employees and, consequently, to different processing times of the tasks.

### 3.2. Controllable Processing Times (CPT)

In this paper, we assume that processing times depend on the resources (or employees, as we would use both terms as equivalent in this paper) assigned. Such type of processing times has been studied in the literature as a function of the resources allocated and of the experience of the workers (level of skills). The relationship between time and amount of resources has been mainly analysed under two different approaches, namely, linear and convex (see the review by [12]). In the linear approach (see, e.g., [35]), a linear relationship between the processing times of a task and the resources allocated to this task is assumed. Denoting *p*_*j*_ as the processing time of task *j* and *u*_*j*_ as the number of resources allocated to this task, this relationship can be expressed as *p*_*j*_ = *a* − *b* · *u*_*j*_, with *a* and *b* constants. However, this approach is not well suited to many realistic situations, since according to Belbin [36], there must be *R*_*j*_ an optimal number of employees to be assigned to task *j* in order to achieve maximum workers' efficiency (point [*R*_*j*_, *pd*_*j*_]). Therefore, it is easy to see that the linear approach is not flexible to this concept, at least for values to each side of the efficiency level since the efficiency point of worker must always be in an extreme of the line (see, e.g., Figure 2(a), where the efficiency point is placed for *a*/*b* resources).

In the convex approach it is assumed that there is an inverse relationship between processing times and employees (see, e.g., [37]), where *c* and *W*_*j*_ (normally denoted workload of the task *j*) are constants:

(1) $$\begin{array}{c} p_{j}={\left(\frac{W_{j}}{u_{j}}\right)}^{c}. \end{array}$$

**Figure 1:**
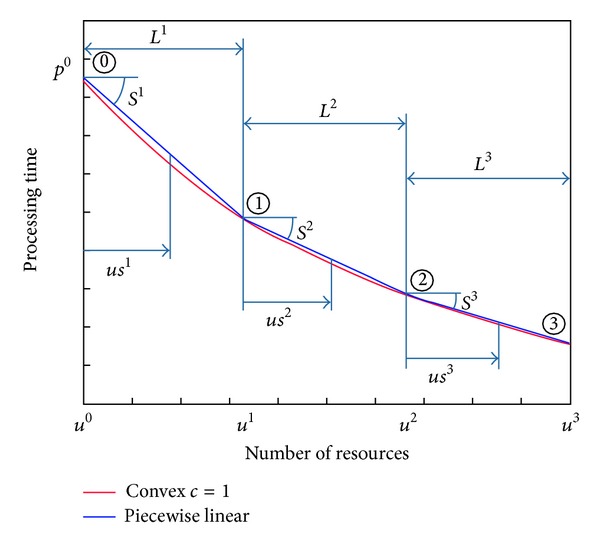
Piecewise linear relationship and convex relationship with *c* = 1. Subscripts are removed for simplicity in the figure.

For *c* = 1, the number of resources and the processing times are inversely proportional (e.g., doubling the employees would cut the processing time by half and vice versa; see the red line of Figure 1). The convex relationship is closer to real life than the linear and it has been used for many actual government and industrial projects [38], distributed communication network, time-sharing computing system, and chemical plant or commercial construction projects [39]. Nevertheless, its nonlinearity would preclude modeling the problem using linear programming. Thus, in this paper a piecewise linear relationship to represent the relationship between processing times and amount of resources assigned to the task is proposed. The relationship is shown in (2), *S*_*j*_^*i*^ being the slope of each section for a task *j* in the piecewise linear relationship and *us*_*j*_^*i*^ the number of employees fulfilled of that task in each section, where section *i* has a length (*L*_*j*_^*i*^). This piecewise linear relationship can be adjusted to the convex relationship (with *c* = 1) as shown in the blue line of Figure 1. This relationship is over the convex relationship representing a safer configuration of processing times and amount of resources:

(2) $$\begin{array}{c} p_{j}=p_{j}^{0}+S_{j}^{1}\cdot {us}_{j}^{1}+\cdots +S_{j}^{k}\cdot {us}_{j}^{k},\\ \text{with}\;\;us_{j}^{i}=L_{j}^{i}\;\forall i<\frac{m}{0}<us_{j}^{m}<L_{j}^{i},\\ S_{j}^{i}=\frac{p_{j}^{i}-p_{j}^{i-1}}{u_{j}^{i}-u_{j}^{i-1}}. \end{array}$$

The superscript for processing times, *p*_*j*_^*i*^, and for the amount of resources, *u*_*j*_^*i*^, denotes that they correspond to point *i*.

**Figure 2:**
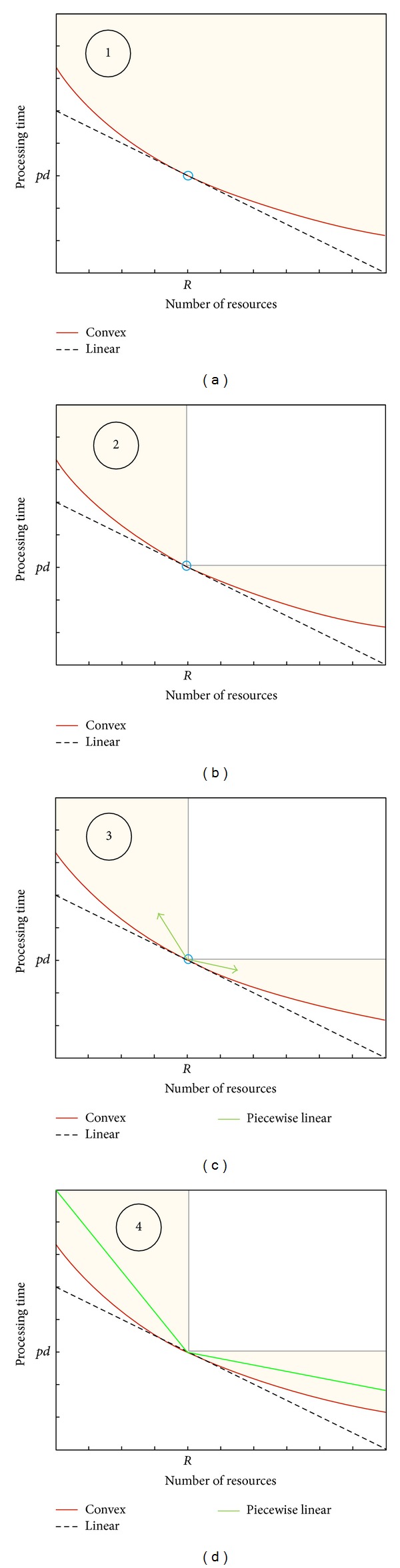
Relation between processing times and number of employees (shaded area).

According to Lanigan [40], the convex relationships between the processing time of a task and the number of employees assigned do not entirely match the reality, and there shall be a penalty due to assigning different numbers of employees. On the one hand, if *u*_*j*_ > *R*_*j*_, then a penalty for communication exists. In contrast (see, e.g., [41, 42]), if *u*_*j*_ < *R*_*j*_ then there is a penalty for lack of specialization [43]. Hence, each feasible point different than the optimum (*R*_*j*_, *pd*_*j*_) must be placed over the convex curve; otherwise many optima would exist and there would be no penalties for under- and overcoverage. The feasible area formed by these points is represented by the light red area in Figure 2(a) (this feasible area is also taken into account by [44, 45]). Moreover, it shall be considered that the processing time of a task decreases when the number of employees increases; otherwise it would make no sense to assign more employees to the tasks. Thus, the feasible area can be reduced by considering that the processing time of a task cannot increase when the number of employees increases (Figure 2(b)). In this way, the feasible relationships between both aspects must be in this area. Therefore, we propose using a piecewise linear relationship with a maximum and minimum possible number of workers on each task. Depending on the maximum and minimum possible number of workers, the slopes of the lines must be different to avoid that both lines are under the convex curve (i.e., in the infeasible region) in any point. The directions of the slope of the piecewise relationships are placed in the feasible region and shown in Figure 2(c). By doing so, linear programming can be used to model and solve the problem. Furthermore, the piecewise relationship is on the safe side with respect to the convex relation, since the piecewise relationship is always over the convex relation.

To define the slopes of the lines, we propose introducing the parameters *kr* and *kl*. The higher these parameters are, the bigger the penalties for under- and overassignment of employees with respect to the optimal value, and consequently the closer the problem to the PSSA with fixed processing times (FPT). In the most extreme case, both problems are the same. The piecewise linear relation left and right of the optimum can be written as follows (see Figure 2(d)):

(3) $$\begin{array}{c} \text{Left:}\;\;p_{j}={pd}_{j}\cdot \left(1+kl\cdot \frac{R_{j}-u_{j}}{R_{j}}\right)\\ \text{Right:}\;\;p_{j}={pd}_{j}\cdot \left(1-\frac{u_{j}-R_{j}}{kr\cdot R_{j}}\right). \end{array}$$

Analogously, let us now consider *R*_*j*_ − *u*_*j*_ as *h*_*j*_^+^ the undercoverage of task *j* and *u*_*j*_ − *R*_*j*_ as the overcoverage of task *j*, *h*_*j*_^−^:

(4) $$\begin{array}{c} \text{Left:}\;\;p_{j}=pd_{j}\cdot \left(1+kl\cdot \frac{h_{j}^{+}}{R_{j}}\right)\\ \text{Right:}\;\;p_{j}=pd_{j}\cdot \left(1-\frac{h_{j}^{-}}{kr\cdot R_{j}}\right). \end{array}$$

### 3.3. Formulation of the Model

#### 3.3.1. Proposed Integer Linear Programming Model

In the previous section, we have presented a mechanism for formulating a realistic relationship between the processing times of the task and the number of employees assigned. Furthermore, this relationship can be embedded into a piecewise linear function; therefore a linear programming model can be formulated for the PSSA. In this section, a formal description of this model is given.

*Data*

 
*r*_*j*_: earliest starting time (release time) of task *j*.
 
bej{1if  the  employee  e  can  perform  task  j0otherwise.iiiiiiiiiiiiiiiiiiiiiiiiiiiiiiiiiiiiiiii
 
prij{1if  task  i  precedes  task j0otherwise.iiiiiiiiiiiiiiiiiiii
 
*R*_*j*_: optimal number of employees of task *j*.
 
*pd*_*j*_: processing time of task *j* for the optimal number employees *R*_*j*_.
 
*LV*_*j*_: maximum allowed undercoverage for task *j*.
 
*UV*_*j*_: maximum allowed overcoverage for task *j*.
 
*kd*,  *kn*: constants related to the slopes of the lines defining the processing times.

*Variables*

 
xjt{1if  task  j  starts  in  period  t0otherwise.iiiiiiiiiiiiiiiiiiiiiiii
 
yejt{1if  employee  e  performs  task  j  during  period  t0otherwise.iiiiiiiiiiiiiiiiiiiiiiiiiiiiiiiiiiiiiiiiiiiiiiiiiiiiiii
 
*h*_*j*_^+^: undercoverage in task *j*.
 
*h*_*j*_^−^: overcoverage in task *j*.
 
*p*_*j*_: processing time of task *j*.

*Auxiliary Variables *

 
zjt{1if  task  j  is  being  processed  at  the  begin  of  period  t0otherwise.iiiiiiiiiiiiiiiiiiiiiiiiiiiiiiiiiiiiiiiiiiiiiiiiiiiiiiiiiiiiii
 
*st*_*j*_: starting time of task *j*.
 
*ct*_*j*_: completion time of task *j*.
 
*C*_max⁡_: project makespan.
 
*ah*_*jt*_: auxiliary variable. It is used to enforce that the constraint set (18) is satisfied if *z*_*jt*_ is zero since the auxiliary variable takes the value of the over- or undercoverage.

*Model*

(5) $$\begin{array}{c} \mathrm{Min}C_{\max} \end{array}$$

(6) $$\begin{array}{c} \overset{T}{\underset{t=1}{\sum }}x_{jt}=1\;\forall j=1,\ldots ,J \end{array}$$

(7) $$\begin{array}{c} st_{j}=\overset{T}{\underset{t=1}{\sum }}x_{jt}\cdot t\;\forall j=1,\ldots ,J \end{array}$$

(8) $$\begin{array}{c} ct_{j}=st_{j}+p_{j}-1\;\forall j=1,\ldots ,J \end{array}$$

(9) $$\begin{array}{c} st_{j}\geq r_{j}\;\forall j=1,\ldots ,J \end{array}$$

(10) $$\begin{array}{c} C_{\max}\geq ct_{j}\;\forall j=1,\ldots ,J \end{array}$$

(11) yejt≤bej·zjt ∀e=1,…,E, ∀j=1,…,J, ∀t=1,…,T

(12) $$\begin{array}{c} st_{j}-st_{i}\geq p_{i}\cdot pr_{ij}-N\cdot \left(1-pr_{ij}\right)\;\forall i,j \end{array}$$

(13) ∑e=1Eyejt+ahjt+(hj+−hj−)=Rj·zjt∀j=1,…,J,  ∀t=1,…,T

(14) $$\begin{array}{c} ah_{jt}\leq \left(1-z_{jt}\right)\cdot N\;\forall j=1,\ldots ,J,\;\forall t=1,\ldots ,T \end{array}$$

(15) ahjt≥−(1−zjt)·N ∀j=1,…,J, ∀t=1,…,T

(16) $$\begin{array}{c} h_{j}^{+}\leq LV_{j}\;\forall j=1,\ldots ,J \end{array}$$

(17) $$\begin{array}{c} h_{j}^{-}\leq UV_{j}\;\forall j=1,\ldots ,J \end{array}$$

(18) ∑j=1Jyejt≤1 ∀e=1,…,E, ∀t=1,…,T

(19) pj≤12+pdj·(1−hj−kr·Rj+kl·hj+Rj)∀j=1,…,J

(20) pj>−12+pdj·(1−hj−kr·Rj+kl·hj+Rj)∀j=1,…,J

(21) $$\begin{array}{c} \overset{T}{\underset{t=1}{\sum }}z_{jt}=p_{j}\;\forall j=1,\ldots ,J \end{array}$$

(22) $$\begin{array}{c} z_{jt}\geq x_{jt}\;\forall j=1,\ldots ,J,\;\forall t=1,\ldots ,T \end{array}$$

(23) 1−zjt≥t−ctjT ∀j=1,…,J, ∀t=1,…,T

(24) $$\begin{array}{c} 1-z_{jt}\geq \frac{st_{j}-t}{T}\;\forall j=1,\ldots ,J,\;\forall t=1,\ldots ,T \end{array}$$

(25) zjt,xjt∈{0,1}, ahjt  free ∀j=1,…,J, ∀t=1,…,T

(26) yejt∈{0,1} ∀e=1,…,E, ∀j=1,…,J, ∀t=1,…,T

(27) $$\begin{array}{c} ct_{j},st_{j},h_{j}^{+},h_{j}^{-},p_{j}\geq 0\;\forall j=1,\ldots ,J \end{array}$$

(28) $$\begin{array}{c} C_{\max}\geq 0. \end{array}$$

Objective function (5) minimizes the project makespan. Constraint set (6) guarantees that each task must start exactly once. Constraint sets (7) and (8) define the starting and completion times of each task, respectively. Constraint set (9) assures that a task cannot start before its release time. Constraint set (10) serves to obtain the makespan. Constraint set (11) ensures with *b*_*ej*_ = 0 that an employee cannot perform a task if he/she does not possess the skills required. Moreover, the variable *z*_*jt*_ is added to the constraint to preclude work at a time when the task is not being processed. Constraint set (12) establishes the precedence relationships. Constraint set (13) defines over- and undercoverage for each task at each period. The auxiliary constraint sets (14) and (15) ensure that constraint set (13) is satisfied for every period. Constraint sets (16) and (17) limit the maximum possible under- and overcoverage, respectively. Constraint set (18) enforces that an employee can work at most in one task at each period. Constraint sets (19) and (20) are used to determine the CPT. Constraint set (21) forces *z*_*jt*_ to be 1 if and only if task *j* is active. Constraint set (22) ensures that task *j* must be performed (*z*_*jt*_ = 1) whenever it starts (*x*_*jt*_ = 1). Constraint sets (23) and (24) model the nonpreemptive assumption: (23) determines that a task cannot be performed (*z*_*jt*_ = 0) after its completion time, and (24) states that it cannot be performed (*z*_*jt*_ = 0) before its starting time. Equations (25), (26), (27), and (28) define the variables employed in the model.

#### 3.3.2. Extension 1: Limitation of Moves

The model presented above allows moving the staff from a task to another. These moves can be detrimental for the service company since they may involve setup and learning times as well as instability in the team composition that performs each task. This limitation of moves can be easily incorporated in the model adding the following two constraints:

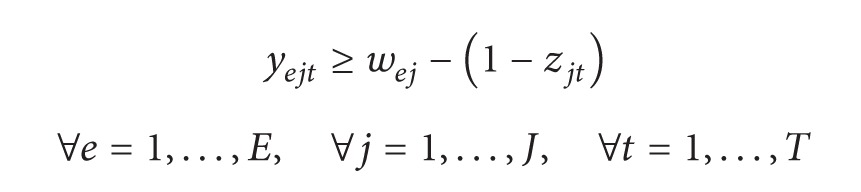
(29)

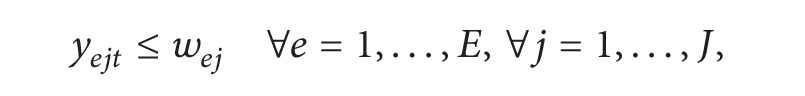
(30)
where *w*_*ej*_ yields 1 if the employee *e* is assigned to the task *j*, 0 otherwise. Constraints (29) and (30) force that an employee have to be assigned to the task during the whole duration of the task when *w*_*ej*_ is equal to 1.

#### 3.3.3. Extension 2: Generic Piecewise Linear Relationship

In Section 3.2 a piecewise linear relationship with two sections was presented to model the relationship between the processing time of a task and the amount of resources assigned to such task. In order to include a generic piecewise linear relationship with* k* sections, the constraints (31)–(38) must replace the constraints (19)-(20):

(31) $$\begin{array}{c} p_{j}=p_{j}^{0}+\overset{k}{\underset{i=1}{\sum }}S_{j}^{i}\cdot us_{j}^{i}\;\forall j=1,\ldots ,J \end{array}$$

(32) $$\begin{array}{c} us_{j}^{i}\leq sa_{j}^{i}\cdot L^{i}+sl_{j}^{i}\cdot L^{i}\;\forall i=1,\ldots ,k,\;\forall j=1,\ldots ,J \end{array}$$

(33) $$\begin{array}{c} us_{j}^{i}>\left(sa_{j}^{i}-1\right)\;\forall i=1,\ldots ,k,\;\forall j=1,\ldots ,J \end{array}$$

(34) $$\begin{array}{c} us_{j}^{i}<\left(2-sa_{j}^{i}\right)\cdot L^{i}\;\forall i=1,\ldots ,k,\;\forall j=1,\ldots ,J \end{array}$$

(35) $$\begin{array}{c} us_{j}^{i}\geq sl_{j}^{i}\cdot L^{i}\;\forall i=1,\ldots ,k,\;\forall j=1,\ldots ,J \end{array}$$

(36) $$\begin{array}{c} \overset{k}{\underset{i=1}{\sum }}sa_{j}^{i}=1\;\forall j=1,\ldots ,J \end{array}$$

(37) $$\begin{array}{c} sa_{j}^{i}\leq sl_{j}^{m}\;\forall j=1,\ldots ,J,\;\forall i,\frac{m}{m}<i \end{array}$$

(38) $$\begin{array}{c} 1-sa_{j}^{i}\geq sl_{j}^{m}\;\forall j=1,\ldots ,J,\;\forall i,\frac{m}{m}\geq i, \end{array}$$

where *sa*_*j*_^*i*^ is an auxiliary variable yielding 1 only in the section *i*, where *us*_*j*_^*i*^ is over 0 and lower than *L* ^*i*^ (represented by the constraints (33)-(34), (36)). Generic piecewise linear relationship is defined in constraint (31). *sl*_*j*_^*m*^ is introduced to define with 1 the sections with *us*_*j*_^*i*^ = *L* ^*i*^, 0 otherwise (constraint (35)). Finally, constraint (32) delimits the upper value of the variable *us*_*j*_^*i*^ and the constraints (37)-(38) establish *sl*_*j*_^*m*^ = 1 in each section *m* preceding the section, where *sa*_*j*_^*i*^ = 1, *sl*_*j*_^*m*^ = 0 otherwise. Thereby, any piecewise linear relationship could be considered in the model by means of these constraints. To adjust this relationship to the convex relationship with *c* = 1, it is necessary only define the parameter *S* ^*i*^ replacing the term *p*(*u*) by *k*/*u* in (2) (see the slope in (39) assuming that the length *L* ^*i*^ = *L* = (*UL* − *LV*)/*k* for each section *i*):

(39) $$\begin{array}{c} S^{i}=\frac{\left(k/\left(LV+L\cdot i\right)\right)-\left(k/\left(LV+L\cdot \left(i-1\right)\right)\right)}{L}. \end{array}$$

### 3.4. Additional Models

#### 3.4.1. Model with Fixed Processing Times (FPT)

In this section, the PSSA problem without CPT is analyzed. The goal is to compare the PSSA model with CPT (PSSA-CPT in the following) with the model with FPT (PSSA-FPT model). To the best of our knowledge, it is the first time that the PSSA problem is solved with processing times depending on the number of employees allocated, so it is relevant to assess the benefits of the assumption. Both models are similar, but there are two main changes. First, in the PSSA-FPT model, processing times do not depend on the number of employees assigned (i.e., this number is fixed and equal to the optimum). Second, over- and undercoverage are not allowed in this model; that is, *h*_*j*_^+^-, *h*_*j*_^−^-, and *ah*_*jt*_-related constraints are not taken into account. The constraint sets (13)–(17), (19)-(20) are then replaced by

(40) $$\begin{array}{c} \overset{E}{\underset{e=1}{\sum }}y_{ejt}=R_{j}\cdot z_{jt}\;\forall j=1,\ldots ,J,\;\forall t=1,\ldots ,T. \end{array}$$

Thus, the PSSA-FPT model consists of the constraint sets (6)–(12), (18), and (21)–(28), where the above *p*_*j*_ variable is now a data of the problem. Obviously, the PSSA-CPT problem is harder than the PSSA-FPT since it has more variables and constraints. It will be analyzed again in the computational results.

#### 3.4.2. Formulation of the Model Using Discrete Time/Resource Trade-Off Problem (DTRTP) Approach with Staff Assignment

The model presented in this paper could be also modeled using a DTRTP approach adding the staff assignment and the release times constraints. However, the results found by this approach were considerably worse in computational time and in average relative percentage deviation. When both models were compared for each instance presented in Section 5.1 (several instances are shown in Table 3), only 363 optimal solutions were found using the DTRTP approach compared to the 567 of the proposed model and the computational time by the DTRTP approach presented a deviation of 287.1% over our proposed model (the deviation runtime is calculated considering only the instances which were optimal solutions found for both models; the amount of these instances is presented in the column of Table 3 denoted by “Both optimum”) as well, justifying the PSSA-CPT model proposed here.

### 3.5. Complexity of the Problem

The PSSA can be reduced to the identical parallel machine problem with two machines (*P*2 | prec,  1 ≤ *p*_*j*_ ≤ 2, *C*_max⁡_ according to Graham et al. [46]), when *E* = 2, *b*_*ij*_ = *R*_*j*_ = 1, and *LV*_*j*_ = *UV*_*j*_ = *r*_*j*_ = 0 are considered. As this problem is NP-hard [47] and our problem is a generalization of it, the PSSA is NP-hard.

On the other hand, the constraint sets (6)–(23) approximately form a total of 9 · *J* + 8 · *J* · *T* + *E* · *T* + *E* · *J* · *T* + *J* · *J*/2 constraints. Regarding the variables, there are 6 · *J* + 3 · *J* · *T* + *E* · *J* · *T* in the model, where 2 · *J* · *T* + *E* · *J* · *T* are binary (i.e., *x*_*jt*_, *z*_*jt*_, and *y*_*ejt*_). The number of constraints and variables is calculated in Table 4 for some instances of the test beds presented in Section 5.1. Since the PSSA with CPT is an NP-hard problem and since the number of constraints and variables for medium-large size of the problem is very high, exact solvers for integer programming problems have difficulties to find feasible and optimal solutions in such instances and therefore approximate algorithms have to be implemented. In the next section, a simple GRASP algorithm is presented to get feasible solutions in lower computational times.

## 4. A Simple GRASP Heuristic Algorithm

Given the complexity of the problem, a greedy randomized adaptive search procedure (GRASP) is presented in this section to solve the PSSA problem with CPT. GRASP was first introduced by Feo and Resende [48] and it has been widely applied to several combinatorial optimization problems. A review of the application fields of this algorithm is done in Festa and Resende [49]. The algorithm consists of two phases: first a constructive phase is implemented, and then a local search phase is made to find better solutions in the neighborhood. The constructive phase is usually employed to find a feasible solution for the problem, while the local search phase is performed to improve the solution found in the constructive phase. Both phases are repeated until the stopping criterion is reached (see Procedure 1).

**Figure 3:**
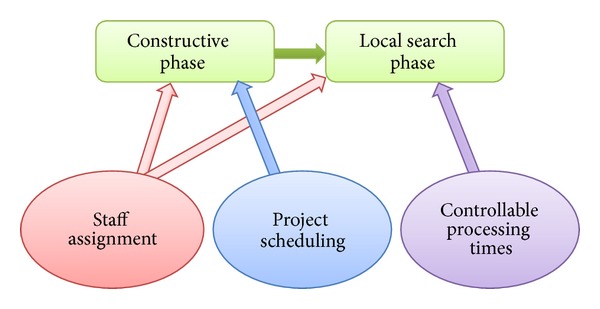
Division of the problem.

In our problem, fixed processing times are considered during the constructive phase. In the local search phase, the task schedule is fixed and more employees are gradually assigned to each task in order to decrease the processing times and thus possibly improving the makespan of the project. In summary, a project scheduling problem is solved in the constructive phase, a CPT problem in the local search phase, while the staff assignment problem is treated in both phases (see Figure 3). In the next subsections the phases are explained in detail.

### 4.1. Constructive Phase

The constructive phase consists in the successive allocation of employees and starting times to tasks. The goal is to assign the tasks as early as possible until all tasks are scheduled. In this phase, both the processing times and the number of assigned employees are kept fixed. The number of employees allocated to each task (*ne*_*j*_) is randomly assigned within the upper and lower limits. The processing times of each task are calculated for the so-obtained number of employees using the expressions shown in Section 3. With respect to the task to be chosen at each step, a restricted candidate list (RCL) is defined based on the tasks' precedence relations. According to these relations, the tasks are classified into sets: set *S*_1_ corresponds to tasks without predecessors, set *S*_2_ is tasks which only have predecessors of set *S*_*k*_, and, in general, set *S*_*i*_ contains tasks with predecessors in sets *S*_*i*−1_, *S*_*i*−2_, …, *S*_1_. The tasks are iteratively inserted in the schedule according to the following procedure. First, the RCL contains all tasks in set *S*_1_. A task is randomly chosen from the RCL to be placed in the schedule. The process is repeated until the RCL is empty. Then, the tasks of set *S*_2_ are moved to. The same procedure is carried out until each task is introduced in the schedule.

Once a task is chosen from the RCL, it has to be placed into the schedule. To do this, the earliest possible start time for the task is calculated. Next, the starting time of the task is fixed to this earliest time and employees (from those having the required skills for the task) are assigned until the number of employees, *ne*_*j*_, is reached. Employees are assigned one by one at random. At each step, the feasibility of the problem is checked. If, at some point, the resulting schedule is not feasible, the starting time of the task is postponed until feasibility is achieved. This process is repeated until the task is definitely inserted in a time period (see function* buildSolution*() in Procedure 3). It is important to note that the processing times are fixed at this phase. The steps of the constructive phase are shown in Procedure 2.

### 4.2. Local Search Phase

After obtaining a schedule of tasks and a staff assignment in the constructive phase, the local search phase aims to improve the solution by reducing the processing times of each task. To do so, tasks are first ordered again at random and then, step by step, the number of employees (*ne*_*j*_) allocated to each task (following this order) is increased. In each step, the processing time of the task is updated and the makespan is analyzed (using the* buildSolution*() function, Procedure 3). If the makespan decreases, then the new solution is kept and the new amount of assigned employees is updated. Next, another employee is assigned to the same task and the objective function is analyzed. This process is repeated until the maximum overcoverage allowed for the task is reached. Then, we continue with the following task in the same way. The local search phase finishes when all tasks have been analyzed. The output of this phase is the best solution found. The pseudocode of this phase is shown in Procedure 4.

## 5. Computational Results

In this section, the problem under consideration is solved using the PSSA-CPT model introduced in Section 3 and the GRASP algorithm presented in Section 4. The PSSA-CPT model is implemented using the software Gurobi Optimizer 4.51 and ILOG CPLEX 12.4 and the GRASP heuristic is coded in C#. The computational experiments were tested on an Intel Core i7-930, 2.8 GHz, 16 GB RAM under Windows 7.

This section is divided into two parts. An explanation of the test bed is presented in Section 5.1 and, next, in Section 5.2, a comparison of the computational results discussed is made, along the following aspects:

- comparison between CPLEX and Gurobi solvers;
- comparison between GRASP algorithm results and exact results;
- impact of CPT on reducing the makespan of the project.

### 5.1. Test Bed

The integer programming problem presented in this paper was solved by the software Gurobi Optimizer 4.5 for instances based on the sets j30 and j60 of the PSPLIB classical instances [50], which are a reference test beds for project scheduling. Each combination of parameters is replicated ten times and the mean value is taken to represent it. In order to adapt the test beds to our problem, additional parameters have to be defined: maximum possible over- and undercoverage (*LV*_*j*_ and *UV*_*j*_) were drawn from uniform distributions [0, *R*_*j*_/2] and [0, *R*_*j*_], respectively; release times were chosen following uniform distributions [0, *T*/6] and the number of employees was the average of the capacities of the 4 employees in the PSPLIB. The test bed used in this paper is similar to the test bed of Drezet and Billaut [17], where an employee was assigned a 70% probability to master a skill. In our paper, it is assigned an 85% probability to avoid infeasibility in the test beds. The parameters *kl* and *kr* are set to 2.5 according to the slope of the piecewise relationships explained in Section 3.2. In total, 960 instances were generated where 10 replicates were obtained for each combination of the parameters in Table 5 (see [51] for a detailed description of the parameters NC, RF, and RS).

### 5.2. Comparison and Analysis of Computational Results

The integer linear programming model was solved in this paper using the Gurobi software and ILOG CPLEX. Firstly, time limit was set to 30 min using Gurobi. The average results out of the 10 replicates for each parameter combination are shown in the 7th and 8th columns of Tables 6 and 7 (for the j30 and j60 instances, resp.). Dashed lines are used in some combination to specify that no solution was found for any replicate. The 9th, 10th, and 11th columns indicate the number of optimal, feasible, and nonfound solutions, respectively, for each combination of parameters found by Gurobi. It can be seen that optimal solutions were achieved for 567 instances from the total of 960 instances, feasible solutions were found for 16 cases, and no solutions before 30 minutes were therefore obtained for 377. The latter represents a huge amount of instances without solution and thus different method of resolution has to be implemented. More specifically, exact solutions were achieved for 325 instances considering 30 tasks in the project, and only in 242 instances with 60 tasks. Using the same time limit than CPLEX, makespan and runtime are shown in the second and third columns. Optimal solutions were found only for 216 from the total of 960 instances (only 13 in the set j60) and feasible solutions for 352 instances and, hence, no solutions were found in 608 instances. This represents worse results as compared to that of Gurobi. With respect to the value of the makespan for the instances where feasible solutions were found, the ARPD (average relative percentage deviation) for CPLEX in these 352 instances was 58.4%, calculated using the expression (41), in contrast to the ARPD of 3.4% found by Gurobi in the 583 feasible solutions:

(41) $$\begin{array}{c} {\text{ARPD}}_{A}=\frac{C_{\max}^{A}-\min\left(C_{\max}^{\text{Gurobi}},C_{\max}^{\text{CPLEX}},C_{\max}^{\text{GRASP}}\right)}{\min\left(C_{\max}^{\text{Gurobi}},C_{\max}^{\text{CPLEX}},C_{\max}^{\text{GRASP}}\right)}. \end{array}$$

Finally, the GRASP heuristic was compared with the exact solutions. The time limit of the GRASP heuristic was set to *J* · *E*/10 seconds; that is, it increases with the difficulty of the problem. Results are shown in Tables 6 and 7. Each row corresponds to a given combination of parameters and the first column indicates the chosen parameters in the format (*j*-NC-RF-RS). The column “*C*_max⁡_^GRASP^” represents the average makespan using the GRASP algorithm for 10 iterations and the column “*t* ^GRASP^” is corresponding computation time (*J* · *E*/10). The mean deviation between the solutions by Gurobi with time limit of 30 min and the solutions by GRASP with time limit of *J* · *E*/10 are shown in the column “ARPD” using the expression (41).

The ARPD of the GRASP algorithm for all problems was 0.38%. Comparing the results of the exact solutions found by Gurobi (567 instances), the deviation of the solutions obtained by the GRASP algorithm is 0.62%, and optimal solutions using GRASP were found in 457 of these 567 instances (80.7% of the instances). Furthermore, feasible solutions were found by GRASP in each instance while feasible solutions were found in 587 instances using Gurobi.

Even better results are obtained when the GRASP algorithm is compared with Gurobi with the same time limit; that is, the time limit *J* · *E*/10 is fixed for both GRASP heuristic and Gurobi. In this case, Gurobi found only optimal solutions in 201 of the total of 960 instances of the test bed while our heuristic found feasible solutions for all instances. Furthermore, the deviation of the heuristic from the optimal solutions was only 0.05% for these 201 instances and optimal solutions were found in 195 instances which represents a 97.01%.

In Section 3.4.1, the interest to compare the PSSA problem with CPT with the PSSA problem with FPT was discussed. The PSSA problem with FPT was solved by Gurobi setting also a time limit of 30 minutes. The makespan and runtime of this problem for each instance are shown in the 11th and 12th columns, respectively. Results of the PSSA-CPT and PSSA-FPT are compared in the 13th column. The objective function of PSSA-FPT represents an upper bound of the function objective of the PSSA-CPT (*C*_max⁡_^PSSACPT^ ≤ *C*_max⁡_^PSSA^), since a solution of PSSA-FPT is always a feasible solution for PSSA-CPT. The difference between the makespan and the runtimes of both models is analyzed. Considering only the instances with optimal solution (567 instances), the makespan decreases by 13.4% using CPT. On the other hand, runtime increased by 160.03%, which may seem an excessive increase in runtimes. However, since the planning horizon is always over 75 days, it seems reasonable that such a long and important decision must be performed carefully, so the runtime increase must not be so significant in that case.

## 6. Conclusions

In this paper, a project scheduling and staff assignment problem has been integrated. As in many real-life cases, the processing times of the tasks depend on the number of employees assigned; CPT were analyzed. There exists a feasible region where the relation of processing times and number of employees can be placed, so a piecewise linear relationship was proposed and included in a linear programming model for the problem in order to better represent the reality considering the penalty for communication in bigger teams and the penalty for lack of specialization in small teams. The consideration of a piecewise linear relationship between the processing times and the amount of resources in the PSSA-CPT provided a large reduction in runtime compared to the adaptation of the DTRTP adding staff assigned.

Different instances of the problem were first solved using the solver Gurobi Optimizer 4.5 and ILOG CPLEX 12.4, the former being more computationally efficient. Furthermore, a comparison between the model with CPT and with FPT was performed to justify the introduction of such CPT. The results obtained highlight an important decrease of the makespan by considering variable processing times. However, due to the complexity of the problem, extremely high computational times were needed for medium-large size instances by Gurobi, justifying the implementation of an approximate algorithm (GRASP). Computational times were greatly decreased there maintaining good ARPDs.

Regarding the future research lines of this paper, different approximate algorithms may be implemented in order to decrease the computational times comparing the results to the GRASP algorithm presented here and with other approximate algorithms employed for similar problems. Finally, a future research line relates to the CPT. In this paper, a piecewise linear relationship between processing times and number of employees (renewable discrete resources) has been introduced in the model. However, different relationships between both variables may be analyzed, such as a convex relationship and hyperbola, in order to adapt the model presented in this paper to continuous renewable and nonrenewable resources.

## Figures and Tables

**Algorithm 1:**
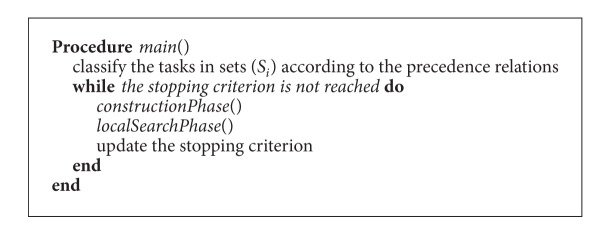
Overall scheme of the GRASP algorithm.

**Algorithm 2:**
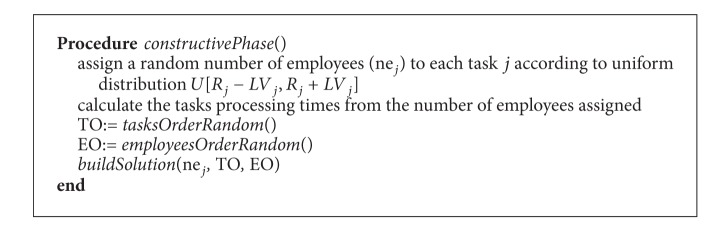
Constructive phase.

**Algorithm 3:**
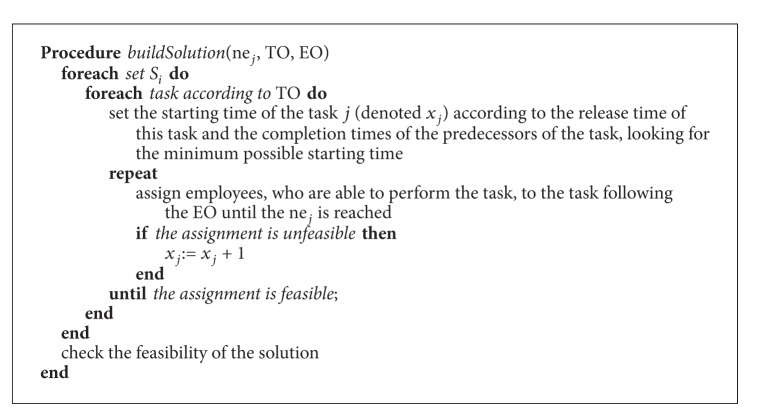
Function *buildSolution*().

**Algorithm 4:**
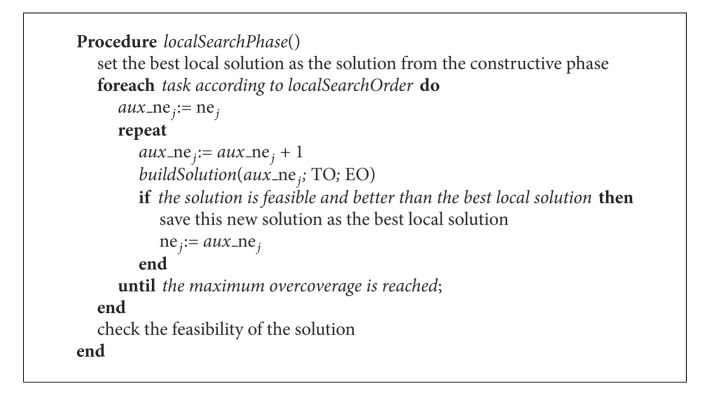
Local search phase.

**Table 1:** Names for the integrated problem.

Reference	Name used for the integrated problem
Bellenguez and Néron [14], Bellenguez-Morineau and N$\overset{´}{\text{e}}$ron [15]	Multiskill project scheduling problem
Brucker and Knust [16], Drezet and Billaut [17]	Project scheduling with labor constraints
Vairaktarakis [18]	Resource-constrained job assignment problem
Valls et al. [19]	Skilled workforce project scheduling
Corominas et al. [20]	Problem of assigning and scheduling a set of tasks to a set of workers
Drexl [21]	Scheduling of project networks by job assignment
Dodin and Elimam [22]	Problem of audit staff scheduling
Heimerl and Kolisch [23], Kolisch and Heimerl [24], Gutjahr et al. [25], and Gutjahr et al. [26]	Project scheduling and staffing
Wu and Sun [27]	Project scheduling and staff assignment

**Table 2:** Summary of related papers.

	Project sched.	Staff assign.	Prec. relations	Release time	Due dates	Preemp.-nonPreemp.	Learning effect	CPT_Skill_	CPT_*N*° of workers_	Move const.	Outsourcing staff	Project selection	Resources per task	Skill	Objective function	Exact methods and solvers	Aprox. alg.
Alfares and Bailey [28]	X	Global	X	X		NP			Data^1^						Cost	^ 2^	Heuristic
Bassett [29]	X	X			X	NP							1	X	Cost	CPLEX	Heuristic
Bellenguez and Néron [14]	X	X	X			NP							n	X	Makespan	Branch and bound	
Bellenguez-Morineau and Néron [15]	X	X	X			NP							n	X	Makespan	Lower bound	
Corominas et al. [20]	X	X	X	X		P	X						1		Makespan	CPLEX	
Dodin and Elimam [22]	X	X	X			NP		Data^1^					1		Cost	Lindo	
Drexl [21]	X	X	X			NP		Data^1^					1		Cost	Branch and bound	Monte Carlo
Drezet and Billaut [17]	X	X	X	X	X	NP				X			n	X	Lateness	CPLEX	Tabu search
Gutjahr et al. [25]	X	X	X	X	X	P	X					X	n	X	Multiobjective	CPLEX	Greedy procedure
Gutjahr et al. [26]	X	X		X	X	P	X					X	n	X	Multiobjective	CPLEX	NSGA-II and P-ACO
Hachicha et al. [30]		X						Data^1^							Cost and preference	Lingo	
Heimerl and Kolisch [31]		X					X	Non Linear						X	Cost		COIN-OR's Ipopt
Kolisch and Heimerl [24]	X	X	X	X	X	NP					X		n	X	Cost	CPLEX	Genetic alg. and tabu search
Valls et al. [19]	X	X	X	X	X	NP		Linear					1	X	Multiobjective		Genetic alg.
Vairaktarakis [18]	X	X	X										1	X	Makespan	Lower bound	Heuristics
Wu and Sun [27]	X	X			X	P	X				X		1		Cost	CPLEX	Genetic alg.
This paper	X	X	X	X		NP			Piecewise linear	X			n	X	Makespan	CPLEX Gurobi	GRASP

^1^Processing times are preprocessed depending on the number of assigned employees or on the level of skills of the assigned employee(s) and then they are introduced in the model as data.

^2^An integer programming model is solved, although the way to do it is not explained.

**Table 3:** Comparison of runtimes between models using DTRTP approach and PSSA with CPT approach.

Instances	DTRTP and staff assignment				PSSA with CPT				Both optimum	Deviation runtime
	#O	#F	#N	Runtime	#O	#F	#N	Runtime		
30-1.5-0.25-0.2	10	0	0	54.0	10	0	0	13.1	10	380%
30-1.8-0.50-0.5	10	0	0	330.2	9	0	1	177.2	9	266%
30-2.1-1.00-1.0	0	0	10	—	9	0	1	—	0	—
60-1.5-0.25-0.2	8	0	2	708	7	0	3	376.6	7	135%
60-1.8-0.50-0.5	1	0	9	—	6	0	4	—	0	—
60-2.1-1.00-1.0	0	0	10	—	5	1	4	—	0	—

Global	363	14	583	376.08	567	20	373	165.26	345	287.1%

**Table 4:** Some examples on the number of constraints and variables.

Instances	Constraints	Variables	Integer variables	Binary variables
30-1.5-0.25-0.2	93120	65701	60661	60480
30-1.8-0.50-0.5	156330	123031	117181	117000
30-2.1-1.00-1.0	237895	204241	198871	198690
60-1.5-0.25-0.2	433887	325801	305461	305100
60-1.8-0.50-0.5	633915	529561	510661	510300
60-2.1-1.00-1.0	1427967	1286641	1262821	1262460

**Table 5:** Test beds.

Parameters	Values
Number of tasks, *j*	30, 60
Network complexity, NC	1.5, 1.8, 2.1
Resource factor, RF	0.25, 0.50, 0.75, 1.00
Strength of the resource, RS	0.2, 0.5, 0.7, 1.0

**Table 6:** Experimental results for 30 tasks.

*j*-NC-RF-RS	CPLEX CPT					Gurobi CPT					Gurobi FPT			GRASP		
	*C*_max⁡_	*t*	#O	#F	#N	*C*_max⁡_	*t*	#O	#F	#N	*C*_max⁡_	*t*	dev.	*C*_max⁡_	ARPD	*T*
30-1.5-0.25-0.2	52.4	224.8	10	0	0	52.4	13.1	10	0	0	58.1	12.6	9.8%	52.4	0.00%	33.3
30-1.5-0.25-0.5	56.8	322.8	9	1	0	54.3	53.9	10	0	0	62.8	19.6	13.5%	54.4	0.20%	40.5
30-1.5-0.25-0.7	62.6	168.4	10	0	0	62.6	15.9	10	0	0	75.4	18.6	17.0%	62.6	0.00%	43.5
30-1.5-0.25-1.0	60.7	133.6	10	0	0	60.7	15.3	10	0	0	69.6	19.4	12.8%	60.7	0.00%	50.1
30-1.5-0.50-0.2	—	1800.0	0	6	4	—	1696.3	1	2	7	—	480.2	—	66.4	0.43%	40.5
30-1.5-0.50-0.5	—	1674.7	3	5	2	—	465.9	9	0	1	67.1	112.7	—	61.5	1.89%	57.7
30-1.5-0.50-0.7	—	1063.1	5	4	1	52.1	277.2	10	0	0	59.0	69.6	11.7%	52.5	0.76%	64.2
30-1.5-0.50-1.0	60.0	297.7	10	0	0	60.0	56.3	10	0	0	68.3	70.3	12.2%	60.0	0.00%	79.9
30-1.5-0.75-0.2	—	1800.0	0	0	10	—	1800.0	0	0	10	—	1489.9	—	72.7	0.00%	44.4
30-1.5-0.75-0.5	—	1800.0	0	8	2	—	1366.1	4	0	6	—	322.1	—	61.6	1.21%	71.5
30-1.5-0.75-0.7	—	1664.6	2	4	4	—	696.8	8	0	2	72.0	104.1	—	64.2	1.95%	76.3
30-1.5-0.75-1.0	83.4	1163.5	6	4	0	—	459.9	9	0	1	64.5	92.2	—	57.2	0.22%	111.7
30-1.5-1.00-0.2	—	1800.0	0	0	10	—	1800.0	0	0	10	—	1800.0	—	78.4	0.00%	51.4
30-1.5-1.00-0.5	—	1800.0	0	3	7	—	1800.0	0	0	10	64.1	359.6	—	61.9	0.00%	75.7
30-1.5-1.00-0.7	—	1653.9	1	4	5	—	1045.4	6	1	3	71.9	137.7	—	66.2	1.44%	90.4
30-1.5-1.00-1.0	—	1319.1	6	1	3	53.0	258.9	10	0	0	59.7	62.8	11.2%	53.0	0.00%	126.1
30-1.8-0.25-0.2	69.8	551.0	9	1	0	63.1	45.3	10	0	0	70.5	47.8	10.5%	63.2	0.17%	32.7
30-1.8-0.25-0.5	60.2	444.6	10	0	0	60.2	47.2	10	0	0	69.1	45.8	12.9%	60.4	0.39%	37.5
30-1.8-0.25-0.7	58.3	370.4	10	0	0	58.3	42.5	10	0	0	66.4	49.9	12.2%	58.3	0.00%	43.2
30-1.8-0.25-1.0	62.9	296.1	10	0	0	62.9	41.8	10	0	0	70.4	47.2	10.7%	62.9	0.00%	48.0
30-1.8-0.50-0.2	—	1800.0	0	6	4	—	1800.0	0	2	8	—	546.5	—	67.6	0.00%	37.5
30-1.8-0.50-0.5	—	1503.1	5	3	2	—	339.5	9	0	1	67.2	108.5	—	59.7	0.91%	55.3
30-1.8-0.50-0.7	—	1116.7	6	3	1	—	254.9	9	0	1	73.9	87.6	—	63.7	0.29%	58.9
30-1.8-0.50-1.0	—	978.2	7	2	1	58.7	114.2	10	0	0	68.2	90.8	13.9%	58.9	0.30%	73.0
30-1.8-0.75-0.2	—	1800.0	0	1	9	—	1800.0	0	0	10	—	1638.6	—	76.2	0.00%	42.6
30-1.8-0.75-0.5	—	1765.8	1	2	7	—	929.5	7	0	3	70.9	130.4	—	64.1	2.29%	65.5
30-1.8-0.75-0.7	—	1644.2	2	5	3	—	419.5	9	0	1	73.9	106.0	—	65.1	1.18%	75.1
30-1.8-0.75-1.0	79.6	973.8	8	2	0	65.2	150.5	10	0	0	74.5	115.6	12.5%	65.5	0.52%	89.2
30-1.8-1.00-0.2	—	1800.0	0	0	10	—	1800.0	0	0	10	—	1800.0	—	96.4	0.00%	47.4
30-1.8-1.00-0.5	—	1800.0	0	3	7	—	1800.0	0	0	10	—	474.0	—	71.6	0.00%	74.2
30-1.8-1.00-0.7	—	1800.0	0	5	5	—	636.2	8	0	2	70.5	170.3	—	64.6	2.55%	87.1
30-1.8-1.00-1.0	—	1664.3	1	4	5	—	631.2	8	0	2	76.9	115.5	—	66.1	1.18%	107.2
30-2.1-0.25-0.2	63.0	823.0	9	1	0	62.2	232.9	10	0	0	71.0	67.5	12.4%	62.3	0.19%	31.5
30-2.1-0.25-0.5	64.0	680.6	9	1	0	64.0	59.7	10	0	0	72.7	60.3	12.0%	64.0	0.00%	36.9
30-2.1-0.25-0.7	66.1	546.3	10	0	0	66.1	47.0	10	0	0	75.6	61.1	12.6%	66.1	0.00%	41.4
30-2.1-0.25-1.0	61.7	455.6	10	0	0	61.7	41.0	10	0	0	73.4	66.2	15.9%	61.7	0.00%	47.1
30-2.1-0.50-0.2	—	1721.4	1	3	6	—	1470.4	2	0	8	—	434.5	—	71.3	0.77%	36.9
30-2.1-0.50-0.5	—	1613.5	3	5	2	—	667.3	8	0	2	77.1	94.5	—	67.7	1.74%	52.0
30-2.1-0.50-0.7	—	1506.5	3	3	4	63.1	367.4	9	1	0	72.3	103.3	12.7%	63.3	0.32%	58.3
30-2.1-0.50-1.0	—	1236.0	5	4	1	69.5	86.5	10	0	0	78.7	101.3	11.7%	69.7	0.30%	70.0
30-2.1-0.75-0.2	—	1800.0	0	0	10	—	1800.0	0	0	10	—	1448.2	—	87.1	0.00%	40.9
30-2.1-0.75-0.5	—	1787.1	1	3	6	—	728.3	7	0	3	76.4	124.9	—	70.9	2.08%	60.4
30-2.1-0.75-0.7	—	1800.0	0	5	5	—	762.1	7	0	3	77.8	113.4	—	67.5	0.97%	67.3
30-2.1-0.75-1.0	—	1196.8	6	1	3	66.9	166.2	10	0	0	75.7	92.2	11.6%	66.9	0.00%	91.3
30-2.1-1.00-0.2	—	1800.0	0	0	10	—	1800.0	0	0	10	—	1800.0	—	94.9	0.00%	44.5
30-2.1-1.00-0.5	—	1800.0	0	0	10	—	1800.0	0	1	9	—	1071.9	—	68.2	0.00%	69.1
30-2.1-1.00-0.7	—	1800.0	0	4	6	—	1096.2	6	0	4	75.4	156.1	—	69.6	1.08%	82.0
30-2.1-1.00-1.0	—	1470.8	5	3	2	—	539.7	9	0	1	69.7	111.9	—	61.7	0.28%	106.9

Average *j*30	—	1271.5	203	110	167	—	84.8	325	7	148	—	346.9	—	65.9	0.20%	61.8

**Table 7:** Experimental results for 60 tasks.

*j*-NC-RF-RS	CPLEX CPT					Gurobi CPT					Gurobi FPT			GRASP		
	*C*_max⁡_	*t*	#O	#F	#N	*C*_max⁡_	*t*	#O	#F	#N	*C*_max⁡_	*t*	dev.	*C*_max⁡_	ARPD	*t*
60-1.5-0.25-0.2	—	1800.0	0	1	9	—	803.6	7	0	3	109.9	246.5	—	100.3	0.30%	79.4
60-1.5-0.25-0.5	—	1800.0	0	3	7	96.7	434.0	10	0	0	107.4	198.2	10.0%	96.9	0.23%	112.4
60-1.5-0.25-0.7	—	1690.5	2	0	8	94.4	235.5	10	0	0	107.8	178.8	12.4%	94.9	0.54%	120.8
60-1.5-0.25-1.0	—	1719.7	2	2	6	99.4	101.5	10	0	0	112.8	167.1	11.9%	99.4	0.00%	153.9
60-1.5-0.50-0.2	—	1800.0	0	0	10	—	1670.2	1	0	9	—	1456.3	—	108.3	0.00%	96.8
60-1.5-0.50-0.5	—	1800.0	0	0	10	—	1193.5	7	1	2	109.2	431.5	—	100.6	0.88%	149.7
60-1.5-0.50-0.7	—	1800.0	0	0	10	—	461.2	9	0	1	109.1	220.4	—	97.0	0.10%	193.0
60-1.5-0.50-1.0	—	1800.0	0	0	10	98.5	285.1	10	0	0	108.8	226.2	9.5%	98.7	0.24%	245.3
60-1.5-0.75-0.2	—	1800.0	0	0	10	—	1800.0	0	0	10	—	1800.0	—	128.9	0.00%	113.1
60-1.5-0.75-0.5	—	1800.0	0	0	10	—	1666.8	1	0	9	—	679.4	—	107.0	0.00%	189.9
60-1.5-0.75-0.7	—	1800.0	0	0	10	—	1514.0	5	1	4	—	491.7	—	93.6	0.65%	248.9
60-1.5-0.75-1.0	—	1800.0	0	0	10	146.0	1115.5	7	3	0	108.4	280.5	—	98.1	0.14%	355.2
60-1.5-1.00-0.2	—	1800.0	0	0	10	—	1800.0	0	0	10	—	1800.0	—	139.1	0.00%	131.6
60-1.5-1.00-0.5	—	1800.0	0	0	10	—	1800.0	0	1	9	—	1259.9	—	99.3	0.00%	223.6
60-1.5-1.00-0.7	—	1800.0	0	0	10	—	1698.8	1	0	9	—	1220.5	—	101.5	0.33%	294.6
60-1.5-1.00-1.0	—	1800.0	0	0	10	—	1633.1	4	0	6	104.1	327.4	—	95.0	0.21%	398.6
60-1.8-0.25-0.2	—	1800.0	0	2	8	—	977.8	7	1	2	111.9	270.9	—	103.5	0.89%	73.9
60-1.8-0.25-0.5	—	1800.0	0	1	9	105.2	253.9	10	0	0	116.0	238.9	9.3%	105.2	0.00%	101.0
60-1.8-0.25-0.7	—	1765.7	1	3	6	103.5	132.0	10	0	0	113.9	186.3	9.1%	103.5	0.00%	113.0
60-1.8-0.25-1.0	—	1699.6	1	6	3	95.9	108.9	10	0	0	108.0	176.7	11.2%	95.9	0.00%	139.4
60-1.8-0.50-0.2	—	1800.0	0	0	10	—	1800.0	0	0	10	—	1577.7	—	121.2	0.00%	90.8
60-1.8-0.50-0.5	—	1800.0	0	0	10	—	957.0	6	0	4	—	515.5	—	106.8	0.09%	149.8
60-1.8-0.50-0.7	—	1800.0	0	0	10	—	696.8	8	0	2	116.8	230.8	—	104.7	0.09%	171.4
60-1.8-0.50-1.0	—	1800.0	0	0	10	97.9	316.4	10	0	0	110.1	189.8	11.1%	98.1	0.19%	209.2
60-1.8-0.75-0.2	—	1800.0	0	0	10	—	1800.0	0	0	10	—	1800.0	—	135.3	0.00%	105.8
60-1.8-0.75-0.5	—	1800.0	0	0	10	—	1737.1	1	1	8	—	1124.3	—	109.2	0.00%	176.7
60-1.8-0.75-0.7	—	1800.0	0	0	10	—	998.9	6	1	3	116.8	327.8	—	106.5	0.20%	211.0
60-1.8-0.75-1.0	—	1800.0	0	0	10	—	906.5	7	2	1	121.6	242.1	—	109.2	0.77%	282.6
60-1.8-1.00-0.2	—	1800.0	0	0	10	—	1800.0	0	0	10	—	1800.0	—	159.7	0.00%	117.2
60-1.8-1.00-0.5	—	1800.0	0	0	10	—	1800.0	0	0	10	—	1539.2	—	124.5	0.00%	207.5
60-1.8-1.00-0.7	—	1800.0	0	0	10	—	1729.2	1	0	9	—	942.2	—	109.7	0.00%	271.1
60-1.8-1.00-1.0	—	1800.0	0	0	10	—	1291.7	8	0	2	120.6	298.3	—	107.3	0.79%	321.6
60-2.1-0.25-0.2	—	1800.0	0	1	9	—	566.9	9	0	1	123.1	215.5	—	111.3	0.31%	73.3
60-2.1-0.25-0.5	—	1534.7	4	1	5	101.6	204.3	10	0	0	112.1	184.5	9.4%	101.7	0.09%	99.2
60-2.1-0.25-0.7	—	1575.9	2	2	6	104.5	151.3	10	0	0	117.1	175.9	10.8%	104.5	0.00%	110.0
60-2.1-0.25-1.0	—	1682.8	1	4	5	103.1	95.8	10	0	0	115.9	157.8	11.0%	103.1	0.00%	130.4
60-2.1-0.50-0.2	—	1800.0	0	0	10	—	1800.0	0	0	10	—	1659.2	—	121.9	0.00%	87.2
60-2.1-0.50-0.5	—	1800.0	0	0	10	—	1417.8	4	0	6	—	558.0	—	105.8	0.85%	139.5
60-2.1-0.50-0.7	—	1800.0	0	0	10	—	621.6	8	0	2	116.2	221.2	—	106.5	0.52%	155.1
60-2.1-0.50-1.0	—	1800.0	0	0	10	108.3	233.5	10	0	0	122.0	192.4	11.2%	108.6	0.27%	195.4
60-2.1-0.75-0.2	—	1800.0	0	0	10	—	1800.0	0	0	10	—	1800.0	—	146.3	0.00%	101.0
60-2.1-0.75-0.5	—	1800.0	0	0	10	—	1800.0	0	0	10	—	1296.2	—	107.5	0.00%	159.9
60-2.1-0.75-0.7	—	1800.0	0	0	10	—	1741.5	2	0	8	124.7	355.5	—	111.5	0.17%	205.0
60-2.1-0.75-1.0	—	1800.0	0	0	10	—	718.1	8	0	2	114.0	208.5	—	101.9	0.40%	257.3
60-2.1-1.00-0.2	—	1800.0	0	0	10	—	1800.0	0	0	10	—	1800.0	—	157.5	0.00%	113.6
60-2.1-1.00-0.5	—	1800.0	0	0	10	—	1800.0	0	0	10	—	1664.8	—	121.2	0.00%	188.8
60-2.1-1.00-0.7	—	1800.0	0	0	10	—	1800.0	0	1	9	—	1020.6	—	109.0	0.00%	239.2
60-2.1-1.00-1.0	—	1800.0	0	0	10	—	1304.8	5	1	4	120.3	240.8	—	108.3	0.54%	301.7

Average *j*60	—	1780.6	13	26	441	—	1112.0	242	13	229	—	712.4	—	110.1	0.53%	175.1

Global	—	1526.1	216	136	608	—	913.7	567	20	377	—	529.7	—	88.0	0.38%	118.5
